# Predictors of intensive care unit admission and mortality in SARS-CoV-2 infection: A cross sectional study at a tertiary care hospital

**DOI:** 10.1016/j.amsu.2022.104097

**Published:** 2022-07-06

**Authors:** Ahmed Alkhamis, Yousef Alshamali, Wassim Chehadeh, Amar Jasem, Abdulaziz Alsayed Omar, Mohammad Alghounaim, Hussein Elsaaran, Sarah Al-Youha, Sulaiman Almazeedi, Moh A. Alkhamis, Salman Alsabah

**Affiliations:** a Department of Surgery, Faculty of Medicine, Health Sciences Center, Kuwait University, Kuwait; b Department of Surgery, Jaber Al-Ahmad Hospital, Kuwait; c Department of Medicine, Jaber Al-Ahmad Hospital, Kuwait; d Department of Microbiology, Faculty of Medicine, Health Sciences Center, Kuwait University, Kuwait; e Department of Pediatrics, Amiri Hospital, Kuwait City, Kuwait; f Department of Epidemiology and Biostatistics, Faculty of Public Health, Health Sciences Center, Kuwait University, Kuwait

**Keywords:** COVD-19, Mortality, Morbidity, Stool, Intensive care unit

## Abstract

**Background:**

The transmissibility and associated morbidity and mortality of severe acute respiratory syndrome-related coronavirus (SARS-Cov-2), have overwhelmed worldwide healthcare systems, resulting in an urgent need to understand this virus and its associated effects. The aim of our study was to identify patient symptoms, clinical characteristics, laboratory, and radiology findings that are associated with serious morbidity and mortality in COVID-19 patients.

**Methods:**

A cross sectional study was conducted in Jaber Al Ahmad Hospital, the designated COVID-19 center in Kuwait between August 1st, 2020 and January 31st, 2021. The main outcomes measured in this study were to identify variables associated with intensive care unit (ICU) admission, as proxy for serious morbidity, and in hospital mortality.

**Results:**

Two hundred and seventy-six patients were included in the study. Thirty-six (13%) patients were admitted to intensive care unit (ICU) and 33 (12%) patients expired. On multivariate analysis we found having elevated fibrinogen [OR 1.39, 95% CI 1.08–1.64, P = 0.04], low estimated glomerular filtration rate (eGFR) [OR 0.89, 95% CI 0.81–0.95, P = 0.02], and having bilateral patchy lung shadowing [OR 6.68, 95% CI 1.85–15.28, P < 0.01] to be significantly associated with increase odds of ICU admission. Elevated CRP [OR 1.25, 95% CI 1.10–1.98, P < 0.01], low eGFR [OR 0.95, 95% CI 0.90–0.99, P = 0.05] and having ischemic heart disease [OR 7.03, 95% CI 1.60–46.42, P = 0.04] were independently associated with increased odds of mortality.

**Conclusion:**

Certain inflammatory and coagulopathy markers, and having certain lung radiological features, in addition to having medical comorbidities, specifically, ischemic heart disease and renal impairment are key predictors for serious morbidity and mortality in patients infected with COVID-19. These should be incorporated into medical institutes risk assessment tools used by physicians and policy makers to instigate, prioritize, and reprioritize care in patients with COVID-19 and instigate preventative strategy to reduce the impact of future outbreak.

## Highlights

• Understanding risk factors associated with worse outcomes when infected with COVID-19 infection is important.
• A multifactorial model incorporating certain laboratory blood tests and patient clinical characteristics seems to be the best way to predict morbidity and mortality.
• Obesity, Cardiovascular and renal deficiencies are predictors of worse outcomes and so should be prioritized in managing COVID-19 patients.

## 1 Introduction

The rapid emergence of the severe acute respiratory syndrome-related coronavirus (SARS-Cov-2) has brought the world to a standstill. The transmissibility and associated morbidity and mortality of this virus had overwhelmed worldwide healthcare systems around the globe, resulting in an urgent need to understand this virus and its associated effects. Nasopharyngeal (NP) swabs were one of the most used tests to accurately diagnose and screen for coronavirus disease 2019 (COVID-19) [1]. The Centers for Disease Control and Prevention guidance for the disposition of patients with COVID-19, used to advise to only discontinue transmission-based precautions for hospitalized patients with COVID-19 based on negative results obtained for at least 2 sequential respiratory tract specimens collected ≥24 h apart [2]. At present, symptoms resolution and or negative exit NP swabs are used by most countries to end transmission-based precautions. However, data showed that in some patients viral shedding can be higher and more prolong in stool compared to NP swabs [[3], [4], [5], [6], [7]]. It is unclear how significant this prolongs fecal shedding in relation to morbidity and mortality outcomes in COVID-19 patients. So, analyzing the relationship between stool PCR samples at various time periods and clinical outcomes seemed interesting. The aim of our study was to identify patient symptoms, clinical characteristics, laboratory, and radiology findings that are associated with serious morbidity and mortality in COVID-19 patients.

## 2 Material and methods

### 2.1 Ethics

The study was approved by Kuwait Ministry of Health Ethical Review Board (reference number. 2020/1418). During the recruitment process a trained physician obtained informed consent from each patient or their next of kin if the patient was unable to consent.

### 2.2 Sample collection

A cross sectional study was conducted in Jaber Al Ahmad Hospital, the designated COVID-19 center in Kuwait between August 1st, 2020 and January 31st, 2021. All hospitalized adult patients (≥ 18 years old) with a diagnosis of COVID-19, based on the World Health Organization COVID-19: Case Definitions [8], and confirmed by real-time polymerase chain reaction (RT-PCR) done on a respiratory sample, were included. All patients diagnosed with COVID-19 stayed in the hospital until they had resolution of certain symptoms, defined as being afebrile for >72 h and having oxygen saturations equal to or above 94%. Nasopharyngeal swabs were collected by trained physicians following standard sample procurement procedure [9]. Nasopharyngeal samples were collected at baseline only. Stool samples were collected on admission, and at 30 days. To improve compliance with study protocol, participants were offered that their stool sample be collected from their residence if they were discharged before 30 days after admission. The main reasons for not having stool sample on admission was the patient being constipated and leaving the hospital before providing the sample. Stool samples not collected at 30 days were mainly because of loss to follow up despite multiple attempts to contact patients. Collected samples were transferred immediately to the Jaber Innovation Laboratory (JIL) at Jaber Al Ahmad Hospital via iced containers and were frozen at −70 °C until processing. Patients were excluded if they expired on admission, were unwilling or unable to provide consent (via themselves or a proxy), refused to provide nasopharyngeal samples on admission, or refused stool samples at either admission or at 30 days.

### 2.3 Data collection

Data regarding patients’ demographics, baseline characteristics, clinical status, laboratory values, radiological tests, inpatient therapies, in hospital morbidity and mortality were collected from the hospital electronic medical record system at baseline and were prospectively followed (Appendix 1). Data regarding patients respiratory (examples; cough, sputum, sore throat, nose & eye congestion, hemoptysis, and dyspnea), gastrointestinal (GI) (examples; nausea, vomiting, and abdominal pain), other/constitutional (examples; fever, headache, loss of smell, loss of taste, lethargy, and dysarthria) symptoms were collected via patients interview on recruitment and day 30 after recruitment if patients were still admitted. As for patients who were discharged before 30 days post recruitment, symptoms were collected through self-administered questionnaire delivered to patient smartphone device and or via phone interviews.

### 2.4 Detection of SARS-COV2 in clinical samples

Extraction and nucleic acid purification are performed using MagMAX™ viral/pathopen nucleic acid isolation kit and KingFisher™ Flex system (Thermofisher, MA, USA). COVID-19 real time RT-PCR was performed using Applied Biosystems™ 7500 Fast RT-PCR system (Thermofisher, MA, USA) as described previously [10].

### 2.5 Outcome measures

The main outcomes measured in this study were to identify variables associated with the need for intensive care unit (ICU) admission, as proxy for serious morbidity, and in hospital mortality.

### 2.6 Statical analysis

Univariate and multivariate statistical analyses were performed using the R statistical software package [11]. We used the random forest algorithm implemented in the MissForest R package [12] to impute missing data. Our Univariate statistical analysis included the chi-square, Fisher's exact, two-sample test and Mann-Whitney tests to assess the significant individual relationships between the risk factors, on one side, and admission to the ICU as well as mortality on the other. A p-value of 0.1 was set as a threshold for including the risk factors for the subsequent multivariable logistic regression analysis. We used the likelihood ratio test and smallest Akaike information criterion to select the final models in a backward elimination approach. We assessed confounding using the 10% threshold change in the regression coefficient approach, while the statistical significance of the likelihood ratio test was used to evaluate all possible two-interactions. Finally, we used the Hosmer–Lemeshow goodness-of-fit statistic to assess the fit of the final models to the data.

The study was compliant as per the STROCSS criteria [52] and is registered with Research Registry registration ID – researchregistry8038 which is accessible using this link https://www.researchregistry.com/browse-the-registry#home/registrationdetails/62b5eaf6e6bd1a001e56f026/

### Funding

A research grant (grant number: CORONA PROP 56) was awarded by Kuwait Foundation for the Advancement of Science (KFAS), government non-profit organization to aid in data collection, purchasing statistical software, database packages, equipment and materials used in the lab for nasal and stool PCR analysis.

### 2.7 Registration of research studies

1. Name of the registry: researchregistry
2. Unique Identifying number or registration ID: researchregistry8038
3. Hyperlink to our study registration: https://www.researchregistry.com/browse-the-registry#home/registrationdetails/62b5eaf6e6bd1a001e56f026/

### 2.8 Patient and public involvement in research

Not involved.

## 3 Results

Two hundred and seventy-six patients were included in the study. Most patients were Kuwaiti nationals. Thirty-six (13%) patients were admitted to ICU and 33 (12%) patients expired. Stool PCR was collected from 197 patients at day 1, and 165 patients at day 30 from the date of COVID-19 diagnosis. Of 197 patients stool samples collected at day one, 141 (71.6%) patients had positive stool PCR. Of 165 patients stool samples collected at day 30, 18 (10.9%) patients had positive stool PCR.

### 3.1 ICU admission (univariate analysis)

The mean age of patients who were admitted to ICU was 53-year-old. Most of them were obese with a mean body mass index (BMI) of 32 kg/m^2^ (Table 1.). We found that having abnormal chest x-ray findings on admission, specifically bilateral patchy shadowing of the lungs, was significantly associated with ICU admission (Table 1.).

**Table 1:** Summary statistics and univariate analysis of baseline characteristics of enrolled patients (N = 276). Significant p-values are boldfaced.

Characteristic	Admitted to the ICU (n = 36; 13.04%)	P-value	Died (n = 33; 11.96%)	P-value
*Demographics*				
Age (years; mean; ± SD)	53.23 (±0.87)	0.31	53.23 (±0.87)	0.01*
BMI	32.02 (±0.42)	0.44	32.02 (±0.42)	0.44
Sex (% Male)	20 (55.56%)	0.13	17 (51.52%)	0.34
*Clinical symptoms upon admission*				
Fever	27 (75.00%)	0.69	25 (75.76%)	0.63
Cough	21 (58.33%)	0.53	20 (60.61%)	0.76
Sputum production	3 (8.33%)	0.34	4 (12.12%)	0.83
Short of breath	19 (52.78%)	0.06	18 (54.55%)	0.04*
Sore throat	0 (0.00%)		2 (6.06%)	0.131
Nasal congestion	0 (0.00%)		3 (9.09%)	0.13
Eye congestion	0 (0.00%)	0.01*	1 (3.03%)	0.22
Headache	5 (13.89%)	< 0.01*	5 (15.15%)	< 0.01*
Lethargy	4 (11.11%)	< 0.01*	5 (15.15%)	< 0.01*
Nausea	9 (25.00%)	< 0.01*	9 (27.27%)	< 0.01*
Vomiting	8 (22.22%)	0.96	6 (18.18%)	0.57
Diarrhea	5 (13.89%)	< 0.01*	4 (12.12%)	< 0.01*
Abdominal pain	0 (0.00%)		2 (6.06%)	< 0.01*
Arthralgias and myalgias	7 (19.44%)	< 0.01*	8 (24.24%)	< 0.01*
Anosmia	0 (0.00%)		1 (3.03%)	< 0.01*
Loss of taste	4 (11.11%)	0.18	3 (9.09%)	0.11
Chest X-Ray		< 0.01*		< 0.01*
Normal	1 (2.78%)		0 (0.00%)	
Ground glass opacity	0 (0.00%)		0 (0.00%)	
Local patchy shadowing	1 (2.78%)		1 (3.03%)	
Bilateral patchy shadowing	34 (94.44%)		32 (96.97%)	
Interstitial abnormalities	0 (0.00%)		0 (0.00%)	
*Positive cases by PCR*				
Stool PCR at Day 1	7 (19.44%)	< 0.01*	7 (21.21%)	< 0.01*
Stool PCR at Day 30	3 (8.33%)	0.62	4 (12.12%)	0.16
*Comorbidities*				
None	11 (30.56%)	0.52	10 (30.30%)	0.53
Hypertension	13 (36.11%)	0.97	12 (36.36%)	0.99
Ischemic heart disease	5 (13.89%)	0.15	6 (18.18%)	< 0.01*
Dyslipidemia	5 (13.89%)	0.77	5 (15.15%)	0.95
Hypothyroidism	3 (8.33%)	0.85	2 (6.06)	0.72
Diabetes		0.18		0.11
Type 1	14 (38.89%)		14 (42.42%)	
Type 2	2 (5.56%)		2 (6.06%)	
Chronic renal failure	4 (11.11%)	< 0.01*	4 (12.12%)	< 0.01*
*Laboratory values at admission*				
Hemoglobin (g/L; mean ± SD)	120.19 (±3.35)	0.07	121.57 (±3.94)	0.19
Platelet (10^9^/L; mean ± SD)	209.33 (±13.44)	0.13	216.03 (±12.82)	0.33
Fibrinogen (g/L; mean ± SD)	6.02 (±0.16)	< 0.01*	6.15 (±0.17)	< 0.01*
D-dimer (ng/mL; mean ± SD)	626.18 (±99.79)	< 0.01*	622.03 (±104.32)	< 0.01*
C-reactive protein (mg/L; mean ± SD)	91.8 (±6.72)	< 0.01*	107.93 (±11.05)	< 0.01*
Estimated glomerular filtration rate (mL/mins/1.73m^2^; mean ± SD)	76.84 (±4.46)		77.26 (±4.21)	
Urea (mmol/L; mean ± SD)	10.74 (±2.06)	< 0.01*	11.06 (±2.21)	< 0.01*
Creatinine (μmol/L; mean ± SD)	100.47 (±14.83)	< 0.01*	100.72 (±15.86)	< 0.01*
Alkaline phosphatase (IU/L; mean ± SD)	124.01 (±15.59)	< 0.01*	121.35 (±17.45)	< 0.01*
Alanine aminotransferase (IU/L; mean ± SD)	43.05 (±5.17)	0.54	42.29 (±5.25)	0.66
Aspartate aminotransferase (IU/L; mean ± SD)	48.91 (±4.85)	0.06	48.03 (±5.19)	0.11
Gamma-Glutamyl transferase (IU/L; mean ± SD)	159.62 (±33.85)	< 0.01*	139.80 (±30.68)	< 0.01*
Total Bilirubin (μmol/L; mean ± SD)	10.39 (±0.59)	0.07	10.85 (±0.61)	< 0.01*
Bilirubin (μmol/L; mean ± SD)	3.19 (±0.32)	< 0.01*	3.19 (±0.29)	< 0.01*
*Treatments*				
Antibiotics	34 (94.44%)	< 0.01*	27 (81.82%)	< 0.01*
Anti-fungal	27 (75.00%)	< 0.01*	21 (63.64%)	< 0.01*
Immunoglobulin	2 (5.56%)	< 0.01*	1 (3.03%)	< 0.01*
Mechanical ventilator	36 (100%)	< 0.01*	28 (84.85%)	< 0.01*
Renal replacement	19 (52.78%)	< 0.01*	16 (48.48%)	< 0.01*
*Mortality*				
Admitted to the ICU	27 (81.82%)	< 0.01*		

SD: standard deviation.

BMI: body mass index.

ICU: Intensive care unit.

PCR: polymerase chain reaction.

Of the 36 patients who were admitted to ICU, 7 patients (19.4%) had positive stool PCR at day 1, and 3 (8.3%) had positive stool PCR at day 30. On univariate analysis it appeared that having a positive stool PCR at day one was significantly associated with ICU admission. However, having a positive stool PCR at day 30 was not significantly associated with ICU admission (Table 1.). The most common medical comorbidity in patients who were admitted to ICU was diabetes (44.4%), followed by hypertension (36.1%), ischemic heart disease (13.9%), dyslipidemia (13.9%) and chronic renal failure (11.1%). Of all medical comorbidities, only renal impairment was significantly associated with ICU admission. Furthermore, analyzing laboratory values on admission we found that having elevated fibrinogen level, elevated D-dimer, elevated C reactive protein (CRP), abnormal renal function markers, and elevated liver enzymes were significantly associated with increased risk of ICU admission. In addition, being on mechanical ventilator, renal replacement therapy, antibiotics, antifungal, and immunoglobulin were also significantly associated with ICU admission (Table 1.).

### 3.2 Mortality (univariate analysis)

Of 36 who were admitted to ICU, 27 (81.8%) died (Table 1). Six patients expired without being admitted to ICU. The mean age of patient who died was 53-year-old. Male patients (51.5%) were slightly more likely to die than female patients and most patients who died were obese, with a mean BMI of 32 kg/m2. (Table 1.). Like the risk of ICU admission, we found having bilateral patchy shadowing on chest X-ray on admission to be significantly associated with increased risk of mortality (Table 1.).

Of all patients who died, 7 patients had positive stool PCR on day one and 4 patients at day 30 after admission. We found that having positive stool sample at day one to be significantly associated with mortality. There was no significant association between having positive stool PCR on day 30 after admission, and risk of mortality (Table 1.). Of all patients who died the majority had diabetes (48.5%), followed by hypertension (36.3%), ischemic heart disease (18.1%), dyslipidemia (15.1%), and chronic renal failure (12.1%). However, we found that only having ischemic heart disease and chronic renal failure were significantly associated with increased risk of death. (Table 1.). In addition, we found that having elevated fibrinogen, elevated D-dimer, elevated CRP, abnormal renal function tests, and elevated liver function enzymes were all significantly associated with increased risk of mortality. (Table 1.). Furthermore, being on mechanical ventilator, renal replacement therapy, antibiotics, antifungal, and immunoglobulin were significantly associated with increased risk of mortality (Table 1.).

### 3.3 Intensive care unit admission and mortality (Multivariate analysis)

On multi-logistic regression analysis, we found that having elevated fibrinogen level on admission increased the odds of ICU admission by 39%, OR 1.39, (95% CI 1.08–1.64, p = 0.04). We also found that having low estimated glomerular filtration rate (eGFR) increased the odds of ICU admission by just over 10%, OR 0.89, (95% CI 0.81–0.95, p = 0.02). Furthermore, it appears that having an abnormal COVID-19 chest X-ray features, specifically bilateral patchy shadowing, to be associated with 6-fold increase in the odds of being admitted to ICU, OR 6.68, (95% CI 1.85–15.28, p **<** 0.01) (Table 2.). Moreover, elevated CRP on admission was associated with 25% increase in the odds of mortality, OR 1.25, (95% CI 1.10–1.98, p **<** 0.1)**.** Like its weight on ICU admission, low eGFR was associated with 5% increase in the odds of mortality (Table 3.). In addition, we found that if patient was admitted to ICU after their diagnosis with COVID-19 there was 13-fold increase in the odds of death (OR 13.46, 95% CI 3.49–51.4, p < 0.01). Of all medical comorbidities, ischemic heart disease appears to cause the most harm. While being infected with COVID-19 was ischemic heart disease was associated with 7-fold increase in the odd of death (OR 7.03, 95%CI, 1.6–46.42, p = 0.04) (Table 3.).

**Table 2:** Multivariate logistic regression model for potential risk factors associated with admission to the ICU.

Risk factor	Odds ratio	95% confidence interval	P-value
Fibrinogen	1.39	1.08–1.64	0.04
e-GFR	0.89	0.81–0.95	0.02
Chest X-Ray			
Normal	Reference		
Lesions	6.68	1.85–15.28	< 0.01*

Hosmer – Lemeshow goodness-of-fit p-value = 0.71.

e-GFR: estimated glomerular filtration rate.

**Table 3:** Multivariate logistic regression model for potential risk factors associated with mortality.

Risk factor	Odds ratio	95% confidence interval	P-value
C-reactive protein	1.25	1.10–1.98	< 0.01*
e-GFR	0.95	0.90–0.99	0.05
Admitted to the ICU			
No	Reference		
Yes	13.46	3.49–51.45	< 0.01*
Ischemic heart disease			
No	Reference		
Yes	7.03	1.60–46.42	0.04

Hosmer – Lemeshow goodness-of-fit p-value = 0.61.

ICU: Intensive care unit.

e-GFR: estimated glomerular filtration rate.

No significant relationship was identified between respiratory, GI, and/or constitutional/other symptoms either on admission or later and the odds of being admitted to ICU and mortality.

## 4 Discussion

Our results showed that being admitted to ICU while being infected with COVID-19 was associated with 13-fold increase in the odds of dying. Our ICU admission rate was 13% and mortality rate was 12%, both in line with other studies. A systemic review of 4203 COVID-19 patients (forty-five studies) reported a pooled ICU admission rate of 10.9% (95%CI, 4.5–19.3), and a pooled mortality rate between 1 and 9.1% [13]. In our study, the majority of patient who were admitted to ICU or died were male and obese. Data do suggest that male sex is independently associated with COVID-19 severity [14,15]. The Center for Disease Control and Prevention (CDC) has issued a list which included BMI >30 as a strong predicter of COVID-19 disease severity [16]. Moreover, a Mexican case control study by Hernández-Garduño reported obesity followed by diabetes and hypertension to be significantly associated with COVID-19 infection and developing severe disease [17].

In our study, elevated fibrinogen level, a proxy for fibrinolysis and disseminated intravascular coagulation, was associated with 39% increase in the odds of ICU admission. Coagulation disorders in COVID-19 patients have been described frequently in the literature. Elevated D-dimer, a proxy for extensive thrombin generation, has been found to be indicative of co-existing thromboembolism in various body systems which can lead to ventilation-perfusion mismatch at respiratory, cardiovascular, and renal levels and subsequent increased risk of organ dysfunction requiring ICU support [18]. As a result, some investigators had successfully been using D-dimer levels to triage care and predict outcomes severity in COVID-19 patients [19]. We believe, fibrinogen should probably be used in the same way. Alteration in other markers of coagulation (thrombocytopenia and prolong prothrombin time) has also been associated with increased risk of death [[20], [21], [22]]. We also found that low eGFR was significantly associated with increased risk of ICU admission and mortality. Uribarri et al. analyzed the data of 758 COVID-19 patients, looking at the impact of kidney function on admission and on their clinical course [23]. They reported that patients with low eGFR was significantly more likely to develop sepsis, develop respiratory failure, and have more in hospital mortality. On multivariate analysis, they also reported renal function to be independent predictor of all-cause mortality. The rational for this might be in part related to their other finding, that patients with low eGFR were less frequently receiving drug therapy for COVID-19 such as hydroxychloroquine or antivirals, likely secondary to concern about their nephrotoxicity. A systemic review of 34 retrospective studies by Kermali et al. also identified worsening of kidney function to be associated with more severe disease [24]. Cheng et al. prospectively analyzed the renal function results of 701 COVID-19 patients, they reported patients with low eGFR were more likely to be severely ill on admission and more likely to expire compared to patients with normal kidney function [25]. The etiology of kidney disease involvement in COVID-19 patients appears to be multifactorial. In early infection phase, the virus infiltrates the lung tissue and begins to proliferate. At this phase, the virus interacts with the renin angiotensin aldosterone system (RAAS) through angiotensin-converted enzyme-2 (ACE2), an enzyme that physiologically counters RAAS activation but also functions as a receptor for COVID-19 [25,26]. ACE2 is a type I membrane protein expressed on the lung, heart, kidney, liver, and the intestine. Recent studies have shown ACE2 expression to be 100-fold higher in the kidney than in the lung [23,27]. This means the virus infects the kidney like the way it does the lung but potentially more aggressively. This also indicates that patient with chronic kidney disease (CKD) might be even more susceptible to getting a complicated COVID-19 infection since they have high activities of RAAS which results in a systemic increased expression of ACE-2 a major entry site for the virus. Another factor that might explain the higher risk of developing symptoms in patients with impaired kidney function, is that it results in the release of systemic toxic cytokine-mediated damage [28,29]. Patient with more aggressive pathophysiology either because of viral and/or host factors, develop during the inflammatory phase of the disease, an exuberant response called “cytokine storm”. This results in generalized aggressive uncontrolled inflammation in many organs, induced by rapid viral replication and cellular damage, viral mediated ACE2 downregulation and shedding, and antibody dependent enhancement. Therefore, massive epithelial and endothelial cell death and vascular leakage trigger more pro-inflammatory cytokines and chemokines that can cause direct kidney damage through apoptosis of renal tubular epithelial cells. This can result in massive leakage of nutrients, proteins and electrolytes including sodium [23]. Finally, the virus might exert direct cytopathic effects on the kidney tissue, which is supported by the detection of polymerase chain reaction fragments of coronavirus in blood and urine in both patients with the 2003 severe acute respiratory syndrome (SARS) and COVID-19 [[30], [31], [32]]. We also found CRP to be significantly associated with 25% increased odds of mortality. Almazeedi et al. retrospectively reviewed 1096 COVID-19 patients in Kuwait and found elevated CRP levels to be associated with 9-fold increase in the risk of ICU admission (OR 9.08, 95%CI, 1.97–49.95, p = 0.015) [33]. Lin et al. reported that in their 217 cohort COVID-19, CRP and lymphocytopenia were independently associated with ICU admission [34]. Luo et al. has retrospectively reviewed 298 patients with COVID-19 reported and reported that age, neutrophil count, platelet count, and CRP were independent predictors of adverse outcome [35]. C-reactive protein was the most significant predictor of all markers and was also an independent discriminator of severe/illness on admission. Henry et al. reported higher CRP values in patient with severe COVID-19 infection in their systemic review of 21 studies (n = 3377) [20]. Data from ship “Diamond Princess” also revealed higher CRP values in patients admitted to ICU [36]. Low CRP levels were also reported by Kronbichler et al. in a systematic review of 34 studies (n = 506), to be predictor of better outcomes [37]. Overall, CRP is a concrete a significant predictor of disease outcomes. Furthermore, it appears that having bilateral patchy lung shadowing on CXR was associated with 6-fold increased risk of ICU admission. Jin et al., in a review of 94 COVID-19 patients, found specific lung parenchymal chest computerized tomography (CT) scan findings to be significant predictors of adverse outcomes [38]. An Italian study found that the diameter of the pulmonary artery to be associated with pulmonary artery hypertension and thus COVID-19 mortality [39]. Another Italian study by Borghesi A et al. used a scoring system which included CXR findings to predict in-hospital mortality in patients with COVID-19 [40]. Lung imaging appears to be, as expected, an important predictor of COVID-19 clinical course.

Ischemic heart disease was a significant independent predictor of in hospital mortality, with 7-fold increase in the odds of death. The American College of Cardiology released a clinical bulletin in March 2020 reporting that mortality was higher among patients with cardiovascular disease (10.5%) compared to diabetes (7.3%), chronic obstructive pulmonary disease (6.3%), hypertension (6%), and malignancy (5.6%) [41]. Furthermore, mounting evidence linked severe COVID-19 infection to cardiac complications like severe systolic dysfunction, fulminant myocarditis, and myocardial infarction [[41], [42], [43], [44], [45], [46], [47]]. Zhou F et al. reported cardiovascular disease to be associated with 21-fold increase in the odds of death [48].

In our aim to identify new clinical, and laboratory markers to help predict COVID-19 outcomes, we looked at stool PCR positivity as promising noninvasive marker. Our study is one of the largest analyzing stool PCR samples at various time periods. We found over 71% of patients diagnosed with COVID-19 on admission (via NP PCR) had positive stool PCR on admission, and 10.9% on day 30. Kim. et al. reported limited serum (2.8%), urine (0.8%), and stool samples (10.1%) positivity on their 74 patients COVID-19 series on admission [49]. They concluded based on this that there is limited non-respiratory transmission of the virus. However, Wang. et al. reported 29% of their 153 COVID-19 patients had positive stool sample on admission [50]. A meta-analysis of 4243 patients, showed the pooled prevalence of positive stool samples was 48.1%, and 70% of patients continued to have positive stool PCR despite having negative NP PCR [4]. Chen et al. found that 66.6% had positive stool sample in their 42 COVID-19 patients at presentation [51]. Lin. et al. reported positive anal swabs in 21.2% of their 217 cohort COVID-19 patients [37]. Viral load was lower in the anal swabs than throat swabs in the early stage of the disease, however, 37% of their patients had persistent positive anal PCR after 1st negative NP PCR, 28% anal PCR remained positive at 21 days, and 19.6% at 30 days. All these reported none-respiratory positive PCR samples at various time periods after 1st diagnosis raise into question the infectivity of these samples, and thus far the literature has lacked concrete answers. In our study we found that having positive stool PCR appeared to correlate well with nasal PCR as predictor of clinical outcomes on admission but not on 30 days follow up. Positive stool PCR at day one but not at day 30 was associated with trends toward increased risk of ICU admission and mortality. Lin et al. also reported that having positive anal PCR on admission, was independently associated with ICU admission, and that the cumulative incidence of ICU admission was higher among patients with positive anal PCR only on admission (26.3% vs. 10.7%, P = 0.006) [37]. Overall, it appears that admission samples rather than follow-up samples can be used as predictive tool for severe outcomes. The mere presence of the virus in the GI tract on follow-up does not seem to correlate with virulence and potentially infectivity and so other variables should be looked at to guide further need of in-hospital precautions. However, we do recognize that our ICU and mortality populations are relatively small and larger sample sizes are needed to make more meaningful conclusions about stool PCR usefulness at various time periods.

### 4.1 Strength & limitations

Our geographical region (Arab, middle east) is understudied in the literature with regards to many pathologies including COVID-19. This study has certain uniqueness as it provides a rare insight into the pathophysiology of the disease in our understudied population in comparison to more studied ones in western and Asia societies.

One major limitation of the study was that it was derived from a single tertiary care center with inherent selection and information bias, hence generalizability of the findings to larger populations might not be representative. Another limitation was a potential recall bias regarding patients’ symptoms at 30 days, since patient were self-administering the follow up questionnaire for COVID-19 symptoms they experienced at 30 days.

## 5 Conclusions and recommendations

A multifactorial model incorporating certain laboratory findings (fibrinogen and CRP levels), radiological findings (Chest X-ray lesions), clinical characteristics such as high BMI, having cardiovascular disease and renal impairment should be used to predict serious morbidity and mortality in patients infected with COVID-19. So, these variables should be incorporated into medical institutes risk assessment tools to instigate, prioritize, and reprioritize care in patients with COVID-19. Furthermore, these should be used as well to guide public health policy makers regarding which medical conditions care should be optimized in tacking this ongoing pandemic and potential future one. Obese patients, patients with cardiovascular disease and renal deficiency should be prioritized in both therapeutic and prevention strategies. These include medical and behavior therapies, medical compliance and health awareness campaigns and instigating surgical interventions such as cardiac revascularization, obesity surgery and renal transplant when indicated. There is evidence that positive stool PCR at the time of diagnosis correlate well with nasal PCR and so can be used as an adjunct to predict disease morbidity and mortality. However, there was no relationship between having positive stool PCR in the body at 30 days after diagnosis and significant outcomes such as serious morbidity and mortality. This can potentially indicate that with time the virus does loss significant proportion of its virulence in the gastrointestinal tract even in sick patients, and further GI sampling is not indicated. This finding alleviates concerns with regards to transmissibility of the virus during the interaction with “symptomatically recovered” COVID-19 patients during GI procedures such as colonoscopy, gastroscopy, and gastrointestinal surgery. However, studies with larger sample size collecting stool sample and clinical data at more frequent and longer time periods are needed to confirm or deny our findings.

## Provenance and peer review

Not commissioned, externally peer reviewed.

## Sources of funding

Research Grant Awarded by the 10.13039/501100003286Kuwait Foundation for Advancement of Science (KFAS), Kuwait City, State of Kuwait Grant number: CORONA PROP 56.

## Ethical approval

The study was approved by Kuwait Ministry of Health Ethical Review Board (reference number. 2020/1418).

## Consent

Written informed consent was obtained from all patients included in the study.

## Author contribution

All authors contributed equally in the protocol, data collection, statistical analysis, and writing.

## Registration of research studies

1. Name of the registry: https://www.researchregistry.com/
2. Unique Identifying number or registration ID: (unique identifying number: researchregistry7324)
3. Hyperlink to your specific registration
  https://www.researchregistry.com/register-now#home/registrationdetails/6181178bc59bee0021a914a7/

## Guarantor

Ahmed Alkhamis ahmed.alkhamis@ku.edu.kw.

## Declaration of competing interest

All authors declare no conflict of interest.

## List of abbreviations

SARS-Cov-2: severe acute respiratory syndrome-related coronavirus
NP: nasopharyngeal
COVID-19: coronavirus disease 2019
RT-PCR: reverse transcription polymerase chain reaction
PCR: polymerase chain reaction
GI: gastrointestinal
ICU: intensive care unit
KFAS: Kuwait Foundation for the Advancement of Science
BMI: body mass index
CRP: C reactive protein
eGFR: estimated glomerular filtration rate
RAAS: renin angiotensin aldosterone system
ACE2: angiotensin-converted enzyme-2
CKD: chronic kidney disease
SARS: severe acute respiratory syndrome
CT: computerized tomography
