# Supplementary material for: Predictors of intensive care unit admission and mortality in SARS-CoV-2 infection: A cross sectional study at a tertiary care hospital
Source: Ann Med Surg (Lond). 2022 Jul 6;80:104097. doi: 10.1016/j.amsu.2022.104097 (PMC9259005; doi:10.1016/j.amsu.2022.104097)
Supplement: Multimedia component 2 [file mmc2.docx]

**Appendix 1.**

**Data collected prospectively during study period**

*a) SARS-CoV-2 symptoms*

Fever

Cough

Short of breath

Sore throat

Nasal congestion

Eye congestion

Headache

Lethargy

Nausea

Vomiting

Diarrhea

Abdominal pain

Arthralgia

Anosmia

Ageusia

*b) Clinical variables*

Heart rate

blood pressure

Respiratory rate

Temperature

Glasgow coma scale (GCS)

Lymphadenopathy

Tonsillar swelling

Throat congestion

Being on antibiotics

Being on antifungals

Being on antivirals

Being on interferon

Being on systemic steroids

Being on Intravenous immunoglobulin

Being oxygen therapy

Being on ventilation

Being on extracorporeal membrane oxygenation (ECMO)

Being on dialysis

Being on vasopressors

Quick sequential organ failure assessment (qSOFA) score

admission to intensive care unit (ICU)

*c) laboratory and radiological variables*

White blood cells

Neutrophils

Lymphocytes

Monocytes

Eosinophil

Hemoglobin

Platelet

Prothrombin time

International normalized ratio

Activate partial thromboplastin time

Fibrinogen

d-dimer

C-reactive protein

Procalcitonin

eGFR

urea

creatinine

magnesium

sodium

potassium

total protein

albumin

alkaline phosphatase

alanine aminotransferase

aspartate aminotransferase

gamma glutamyl transferase

total bilirubin

direct bilirubin

calcium

hba1c

troponin

blood culture

sputum culture

urine culture

stool culture

Stool polymerase chain reaction (PCR)

Nasal PCR

Chest X ray finding

*C) medical comorbidities*

Hypertension

Ischemic heart disease

Dyslipidemia

Hypothyroidism

Immunodeficiency

Inflammatory bowel disease

Lymphoma

Diabetes mellitus

Leukemia

Solid tumor

Peptic ulcer disease

Dementia

Autoimmune deficiency syndrome

Severe or moderate chronic renal failure

Chronic obstructive pulmonary disease

Cerebrovascular accident or transient ischemic attack

Peripheral vascular disease

Liver disease

Myocardial infarction

Congestive heart failure

Connective tissue disease

*D) Demographics*

date of birth

gender

nationality

smoker

height

weight

*e) Adverse events:*

Acute respiratory distress syndrome (ARDS)

Septic shock

Acute renal failure

Disseminated intravascular coagulation

Rhabdomyolysis

Pneumonia

Limb ischemia

Bowel ischemia

others
